# Age-Related Differences in the Association between Plasma High-Sensitivity C-Reactive Protein and Noncalcified or Mixed Coronary Atherosclerotic Plaques

**DOI:** 10.1155/2020/5938957

**Published:** 2020-04-28

**Authors:** Tiewei Li, Ning Chen, Zhengan Liu, Zhiming Shan, Geng Dong, Junmei Yang, Minglu Qi

**Affiliations:** ^1^Zhengzhou Key Laboratory of Children's Infection and Immunity, Children's Hospital Affiliated to Zhengzhou University, Henan Children's Hospital, Zhengzhou Children's Hospital, 33 Longhu Waihuan East Street, Jinshui District, Zhengzhou 450000, China; ^2^General Hospital of Taiyuan Steel (Group) Co., Ltd., 7 Yingxin Street, Jiancaoping District, Taiyuan 030000, China

## Abstract

**Background:**

Previous studies have demonstrated that plasma high-sensitivity C-reactive protein (hsCRP) was the predictor for unstable coronary plaque. Patients with noncalcified plaque (NCP) or mixed plaque (MP) have a higher risk of poor outcomes. However, the association between hsCRP and the presence of NCP or MP (NCP/MP) in old adults remains unclear, and if present, whether there exist differences between young and old adults remain unknown. Thus, the aim of this study was to investigate the role of hsCRP in predicting the presence of NCP/MP and evaluate whether age has any impact on this association.

**Methods:**

A total of 951 subjects were included in this study. Complete clinical and laboratory data were collected. According to the characteristics of the most stenotic plaque, we divided them into 2 groups: calcified plaque (CP) and NCP/MP. Subjects with no plaque were classified as the control group (CR). Subjects with age ≥ 60 years were defined as older adults, and those with age < 60 years were classified as nonelderly people.

**Results:**

Patients with NCP/MP had significantly higher hsCRP level compared with subjects with CR or CP in older adults but not in nonelderly people. The proportion of NCP/MP was significantly increased from 27.0% in the hsCRP < 1.25 mg/L group to 42.7% in the hsCRP > 2.70 mg/L group in older adults. Multiple logistic regression analysis showed that hsCRP was an independent risk factor for the presence of NCP/MP (odds ratio (OR) = 1.093, 95% CI 1.032–1.157, *P* = 0.001) only in older adults.

**Conclusions:**

hsCRP is independently associated with the presence of NCP/MP in older adults but not in nonelderly people. These results suggest the potential significance of hsCRP-lowering regimens in older adults with NCP/MP.

## 1. Introduction

Cardiovascular disease remains the leading cause of morbidity and mortality. Atherosclerosis, characterized by coronary atherosclerotic plaques, is the common cause of coronary artery disease (CAD). According to postmortem studies, 75% of episodes of acute coronary syndromes (ACS) was caused by plaque rupture. Plaque rupture can lead to a rapid release of large amounts of components from the plaque into the blood, which activates the coagulation system to form a thrombus and results in the occlusion of the blood vessels.

Numerous studies have established that atherosclerosis is a chronic inflammatory disease, and there is a fundamental role for inflammation in mediating all stages of this disease from initiation through progression and, ultimately, the thrombotic complications of atherosclerosis. C-reactive protein (CRP) is an acute-phase protein of hepatic origin that increases in response to inflammation and is used as a marker of inflammation. Multiple studies demonstrated that CRP was associated to cardiovascular disease [1–3]. Matsuura et al. reported that CRP was more intense in culprit plaques from patients with unstable angina pectoris than from those with stable angina pectoris, and it was significantly correlated with CD68- (pan macrophage) positive areas [4]. Several studies have shown that high-sensitivity C-reactive protein (hsCRP) was an independent predictor of the severity of coronary artery disease [5, 6]. In addition, hsCRP has also been shown to be an independent predictor for the adverse cardiovascular outcomes of coronary heart disease, myocardial infarction, heart failure, stroke, atrial fibrillation, and chronic obstructive pulmonary disease [7, 8].

Previous studies demonstrated that noncalcified plaque (NCP) and mixed plaque (MP) were prone to rupture and have an association with plaque volume progression [9, 10]. Furthermore, Hou et al. demonstrated that patients with NCP or MP (NCP/MP) had a higher risk of poor outcomes compared with those without coronary plaque or those who had calcified plaques (CPs) [11]. Previous studies have demonstrated that neutrophil-lymphocyte ratio and fibrinogen were the independent predictors of NCP/MP [12, 13]. However, there are few published data about the association between CRP and NCP/MP. Several studies demonstrated that CRP was associated with the instability of coronary plaques [14–16]. Yang et al. reported that CRP was associated with noncalcified coronary arterial plaque [17].

It is well known that aging is an inevitable part of life and unfortunately poses the largest risk factor for cardiovascular disease [18]. Corban et al. [19] reported that age was an independent predictor of plaque area. In addition, there is also a positive association between age and hsCRP [20, 21]. However, there are no published data regarding the effect of age on the relationship between hsCRP and high-risk plaque. Therefore, the aim of this study is to evaluate whether age has any impact on the association between hsCRP and the presence of NCP/MP.

## 2. Materials and Methods

### 2.1. Participants

A total of 951 consecutive subjects were included in this study. All subjects underwent coronary computed tomography angiography (CCTA) due to stable typical or atypical chest pain in the General Hospital of Taiyuan Steel (Group) Co., Ltd. Clinical and laboratory data were collected. Subjects with the following conditions were excluded from this study: (1) subjects without lipid profiles and hsCRP measurements; (2) patients with acute coronary syndrome and other diseases, such as congenital heart disease, New York Heart Association (NYHA) functional class III or IV heart failure, stage C or D of the progression of valvular heart disease according to the 2014 guideline of the American Heart Association/American College of Cardiology (AHA/ACC) for the management of patients with valvular heart disease, hematological disease, cancer, and severe renal or liver disease; and (3) subjects with known factors influencing hsCRP level (infection with fever or use of anti-inflammatory drugs). Subjects were divided into two groups. Subjects with age ≥ 60 year were defined as older adults in Chinese. Nonelderly people were those with age < 60 years. The study protocol complied with the Declaration of Helsinki and was approved by the hospital ethics review board. Written informed consent was obtained from all the participants.

### 2.2. CCTA Data Acquisition and Image Analysis

Scans were performed using a 64-row spiral CT scanner (LightSpeed VCT, GE Healthcare, Milwaukee, WI, USA). The main scanning parameters were as follows: 64 detectors; individual detector width: 0.625 mm; gantry rotation time: 350 ms; tube voltage: 120 kV; electrocardiographically modulated tube current; pitch: 0.16 to 0.22; table feed per rotation: 400 mm; and field of view: 200 to 250 mm. The scans were retrospectively analyzed at the workstation (Deep Blue, ADW4.3, GE Healthcare). According to the atherosclerosis plaque density, plaques were classified as CP, NCP, or MP. Briefly, CP was defined as lesions with attenuation values > 130 HU (measured in Hounsfield Units (HU)). Lesions with attenuation values < 130 HU were classified as NCP. MP had both the CP and NCP components. Participants were grouped according to the characteristic of the most stenotic plaque. Previous studies have shown that patients with NCP/MP had a higher risk of poor outcomes [11, 22]. Thus, NCP and MP (NCP/MP) were combined for analysis.

### 2.3. Cardiovascular Risk Factor Assessment

The traditional risk factors for cardiovascular risk such as hypertension, diabetes mellitus, dyslipidemia, cigarette smoking, alcohol consumption, and family history of coronary heart disease (CHD) were assessed. Hypertension was defined as systolic blood pressure > 140 mm Hg, diastolic blood pressure < 90 mm Hg, or taking antihypertensive medication. Diabetes mellitus was defined as fasting glucose of ≥126 mg/dL, nonfasting glucose of ≥200 mg/dL, or receiving hypoglycemic therapy. Dyslipidemia was defined by medical history or fasting total cholesterol (TC) ≥ 5.18 mmol/L or triglyceride (TG) ≥ 1.70 mmol/L or the use of lipid-lowering medications in order to reduce lipids. Smoking status and alcohol consumption were ascertained by the medical history. Family history of CHD was considered as a history of CHD, myocardial infarction, coronary revascularization, or sudden cardiac death before 55 years of age for the father or 65 years of age for the mother.

### 2.4. Biochemistry

Venous blood samples were obtained at baseline before undergoing CCTA. Serum hsCRP was measured using the Particle Enhanced Immunoturbidimetric Assay (Ultrasensitive CRP kit, Orion Diagnostic, Espoo, Finland) on an Olympus AU5400 analyzer (Olympus Diagnostics, California, USA). The levels of serum total cholesterol (TC), triglyceride (TG), low-density lipoprotein cholesterol (LDL-C), and high-density lipoprotein cholesterol (HDL-C) were measured using an automatic biochemistry analyzer (Olympus Diagnostics, California, USA) and a conventional clinical analytical methods.

### 2.5. Statistical Analysis

Statistical analysis was performed using the statistical package SPSS 21.0 (SPSS Inc., Chicago, Illinois, USA). Continuous variables are expressed as either mean ± standard deviation (SD) or median (interquartile range, (IQR)), according to their distribution. The differences were analyzed by independent-samples *t*-test, one-way ANOVA, or the Mann-Whitney *U* test, as appropriate. Categorical variables were expressed as percentages and were assessed by *χ*^2^ or Fisher's exact tests. Pearson's or Spearman's correlation test was used to examine correlations between two continuous variables when indicated. Multivariate logistic regression analysis was performed to identify the independent risk factors of the presence of NCP/MP. The risk factors were prespecified on the basis of univariate *P* values of <0.10 and previously published literature. A two-sided *P* < 0.05 was considered significant.

## 3. Results

### 3.1. Baseline Clinical Characteristics of Study Subjects

Baseline clinical and laboratory data of the studied population stratified by the characteristics of coronary atherosclerosis plaques are summarized in Table 1. A total of 951 subjects undergoing CCTA were enrolled in this study. The majority of the study population (*n* = 752, 79.1%) had atherosclerotic plaques, and the remaining 199 participants had no coronary plaques which served as control (CR). Among the 752 patients, 388 patients had CP and 364 patients had NCP/MP.

**Figure 1:**
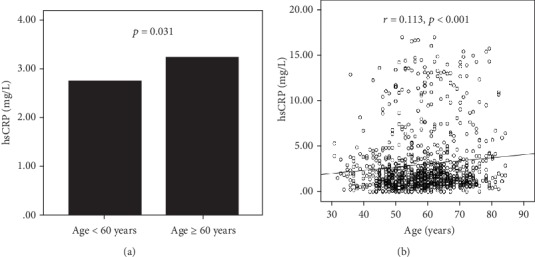
(a) Plasma hsCRP level in older and nonelderly people. (b) The correlation of hsCRP with age. Abbreviations: hsCRP—high-sensitivity C-reactive protein.

Patients with CP or NCP/MP were older and had a higher percentage of males than the subjects without plaques. There was also a higher prevalence of hypertension, diabetes mellitus, dyslipidemia, smoking, and alcohol consumption in the CP and NCP/MP groups compared with the CR group. The laboratory data showed that the levels of TC and LDL-C were significantly lower in the CP and NCP/MP groups compared with the CR group (*P* < 0.05), which might be explained by the higher proportion of patients with CP and NCP/MP using statins (73.4 and 67.3 vs. 31.7%; *P* < 0.001). In addition, the levels of hsCRP showed a progressive increase among the 3 groups. Meanwhile, the level of hsCRP was significantly higher in older adults, and there was a positive association between hsCRP and age (Figure 1).

### 3.2. Changes in Plasma hsCRP Level according to Age-Specific Type of Plaques

Subjects with age > 60 years were defined as older adults in Chinese. In this study, subjects were divided into two groups: the group of older people (age ≥ 60 years) and the group of nonelderly people. The clinical characteristics of the two groups of people are presented in Table 2. In older people and nonelderly people, age, the prevalence of hypertension, dyslipidemia, cigarette smoking, alcohol consumption, and medications taken (including stains, aspirin, calcium antagonists, ARB/ACEI, and beta-blockers) were all significantly different among the three groups, while BMI and the prevalence of diabetes mellitus showed significant differences among the three groups only in older people. Of note, hsCRP level did not differ significantly in subjects with age ≥ 60 years (*P* > 0.05), whereas they did show a progressive increase among the three groups in subjects with age < 60 years.

To further investigate the relationship between hsCRP and NCP/MP, we divided the participants into three groups according to age-specific tertiles of hsCRP (Table 3). Nonelderly people in the third tertile had a higher level of BMI and a higher prevalence of hypertension, while in older people, the percentage of males and the level of TC and LDL-C showed a significant difference among the three groups. Meanwhile, the level of HDL-C was the lowest among the three groups in both nonelderly and older people. Of note, in females, the prevalence of NCP/MP increased significantly from 27.0% in the first tertile to 44.7% in the third tertile (*P* = 0.007), whereas these did not show a significant difference among the hsCRP tertiles in nonelderly people.

### 3.3. Age-Specific Correlation between hsCRP and Other Cardiovascular Risk Factors

To explore the correlation between variables and hsCRP, Spearman's correlation analysis was performed in the study. As shown in Table 4, hsCRP was positively correlated with BMI (*r* = 0.173, *P* < 0.001) and TG (*r* = 0.132, *P* = 0.002) in nonelderly people but not in older people. Meanwhile, the data showed that hsCRP had a negative association with HDL-C in both nonelderly (*r* = −0.258, *P* < 0.001) and older (*r* = −0.203, *P* < 0.001) people. In addition, there was no significant correlation between hsCRP and age and between TC and LDL-C.

### 3.4. Multivariate Analysis to Identify Predictors of the Presence of NCP/MP

Multivariable binary logistic regression analysis was performed to identify potential predictors of the presence of NCP/MP. Variables with *P* < 0.05 in univariate analysis and the previously published literature were included in the model of multivariate analysis including age, sex, BMI, hypertension, diabetes, dyslipidemia, smoking, statin, aspirin, and ARB/ACEI. As shown in Table 5, multivariate analysis showed that hsCRP was the independent predictor of the presence of NCP/MP (odds ratio (OR) = 1.089, 95% CI 1.030-1.152, *P* = 0.003) only in older adults. Furthermore, in older adults, elevated hsCRP level was significantly associated with increased risk of the presence of NCM/MP. Meanwhile, logistic regression analysis showed that hsCRP was also a protective factor of the presence of CP (OR = 0.932, 95% CI 0.882-0.986, *P* = 0.014) in older adults.

## 4. Discussion

NCP and MP defined by CCTA were characterized by low-density material. Several studies have demonstrated that the culprit lesions in patients with ACS were more prone to be NCP, and patients with NCP had increased risk for adverse poor outcomes [10, 11]. Meanwhile, Pundziute et al. reported that MP was more prone to be thin-cap fibroatheroma and the number of MP determined by multislice computed tomography was an independent predictor of acute cardiac events [9]. Hou et al. further demonstrated that patients with NCP/MP had a 3 times higher risk of 3-year major adverse cardiac events, in 5007 outpatients with suspected CAD. So, early identification of NCP/MP is clinically useful [11]. However, CCTA is not suitable for large-scale screening to identify NCP/MP due to the radiation exposure, complicated procedure, and high cost. Therefore, easily measured and cheap circulating biomarkers might provide valuable information.

CRP is an annular, pentameric protein synthesized by the liver in response to factors released by macrophages and fat cells (adipocytes) that circulates in blood plasma [23]. CRP is an acute-phase protein that increases in response to inflammation. Mounting evidence has established the role of CRP in atherosclerosis [24–26]. Histopathological study showed that the expression of CRP was more intense in culprit plaques from patients with UAP than with SAP, and they significantly correlated with CD68-positive areas [4]. Devaraj et al. [27] found that CRP could promote endothelial dysfunction by inducing the release of circulating endothelial cells and endothelial microparticles, which were biomarkers of endothelial dysfunction. CRP further increased the transcytosis of LDL across endothelial cells and LDL retention in vascular walls, which promoted atherosclerosis [28]. In addition, Cimmino et al. [29] reported that CRP induced matrix metalloproteinase-9 (MMP-9) expression and activity in human SMCs in culture; patients presenting with ACS have increased transcoronary plasma levels of MMP-9 and CRP with a significant correlation between these two markers. MMP-9 mainly degrades type IV collagen and fibronectin and contributes to both the formation as well as the destabilization of atherosclerotic plaques [30].

Numerous clinical studies as well as meta-analysis studies have established that CRP was an independent risk factor for cardiovascular disease, including CHD, myocardial infarction, stroke, and other vascular mortality [2, 7, 31]. Furthermore, one study reported that assessment of CRP in people at intermediate risk for a cardiovascular event could help prevent one additional event over the course of 10 years under current treatment guidelines [3]. In addition, Cheng et al. showed that hsCRP was a marker of coronary plaque burden. Even though under high-intensity statin therapy, baseline-to-follow-up change of hsCRP correlated with the change in percent necrotic core volume [16]. Meanwhile, it has been reported that CRP level did significantly increase with age [32] and age was an independent risk factor for high-risk plaque [33]. However, there are few published studies to evaluate whether age has any impact on the relationship between hsCRP and the presence of NCP/MP.

In the current study, we found that the NCP/MP group had the highest level of hsCRP among the three groups. To investigate the effect of age on the relationship between hsCRP and the presence of NCP/MP, subjects were divided into two groups: nonelderly people (age < 60 years) and older adults (age ≥ 60 years). Interestingly, after stratifying by age, we found that the statistical significance of hsCRP among the three groups only existed in older adults but not in nonelderly people. Further analysis showed that the prevalence of NCP/MP showed a progressive increase from the tertile 1 to tertile 3 of hsCRP in older adults but not in nonelderly people. Multivariate analysis showed that hsCRP was the independent predictor of the presence of NCP/MP in older adults.

Of note, these associations were found only in older adults indicating that age might have some impact on the relationship between hsCRP level and the presence of NCP/MP. Firstly, aging is an inevitable part of life and unfortunately poses the largest risk factor for cardiovascular disease [34]. Aging-associated changes could be observed in multiple cell types [35]. Aged ECs showed increased expression of the adhesion molecules (VCAM-1 and ICAM-1) and increased susceptibility to apoptosis [36]. In addition, Song et al. reported that aged VSMCs exhibit a higher secretion of interleukin-6 and upregulation of chemokines CCL2, ICAM-1, and Toll-like receptor 4 (TLR4) compared with young patients, which generated a proinflammatory environment and further promoted atherosclerosis progression [37]. Epidemiological studies showed that the prevalence of coronary plaque increased with age, and in the highest age tertile, there was a 2.5-fold increase in the burden of noncalcified plaque [38], while younger patients had a higher risk for isolated NCP [39]. Secondly, previous studies and our data all showed that there was a positive correlation between age and hsCRP, and older adults had a higher level of hsCRP (data not shown) compared with nonelderly people [32, 40]. In addition, in patients with acute coronary syndrome (ACS), Badran et al. reported that the correlation between hsCRP and the severity of CAD only existed in older groups, which was similar to our results that the association between hsCRP and the presence of NCP/MP only existed in older adults, not in young patients [40]. These phenomena of age-related differences in atherosclerosis and hsCRP level may partly be the reason for the age-related differences in the association between plasma hsCRP and the presence of NCP/MP.

There are several limitations in our study. First, this is a cross-sectional and single-center study for which some inherent biases were unavoidable. Second, we only divided the subjects into the older group (age ≥ 60 years) and the nonelderly group (age < 60 years) and failed to continue grouping by age due to the limited sample size. In addition, the sample size of CR in the elderly is small. The findings of this study need further validation in a large population. Third, serial measurements of hsCRP and changes of the characteristic of plaque might provide more implications and be useful to further explore the dynamic correlation between them. Finally, all of the enrolled subjects were Chinese and had symptoms of chest pain. The findings of this study cannot be applicable for other ethnic groups and the general population.

## 5. Conclusion

To the best of our knowledge, this study demonstrates for the first time that there existed age-related differences in the relationship between hsCRP and the presence of NCP/MP. HsCRP level was higher in older adults than in nonelderly people and showed a progressive increase among the three groups only in older adults. Meanwhile, multivariate analysis showed that hsCRP was independently associated with the presence of NCP/MP in older adults. These findings suggest that the age-related differences should be taken into account in therapeutic approaches to regress NCP/MP by using hsCRP-lowering drugs.

## Figures and Tables

**Table 1:** Baseline characteristics of the study population according to the type of plaques.

Variables	CR (*n* = 199)	CP (*n* = 388)	NCP/MP (*n* = 364)	*P*
Age (years)	51.9 ± 8.8	62.5 ± 9.6	57.7 ± 10.7^a^	<0.001
BMI (kg/m^2^)	24.9 ± 3.5	25.6 ± 3.7	25.7 ± 3.8	0.026
Male, *n* (%)	89 (44.7%)	204 (52.6%)	243 (66.8%)^a^	<0.001
Hypertension, *n* (%)	92 (46.2%)	298 (76.8%)	256 (70.3%)	<0.001
Diabetes, *n* (%)	21 (10.6%)	96 (24.7%)	107 (29.4%)	<0.001
Dyslipidemia, *n* (%)	77 (38.7%)	300 (77.3%)	299 (82.1%)	<0.001
Smoking, *n* (%)	39 (19.6%)	95 (24.5%)	126 (34.6%)^a^	<0.001
Alcohol consumption, *n* (%)	41 (20.6%)	99 (25.5%)	119 (32.7%)^b^	0.005
History of CAD, *n* (%)	27 (13.6%)	39 (10.1%)	41 (11.3%)	0.443
Biochemical parameters				
TC (mmol/L)	4.88 ± 1.04	4.51 ± 1.36	4.58 ± 1.21	0.002
TG (mmol/L)	1.45 (1.02, 2.08)	1.47 (1.08, 2.05)	1.55 (1.15, 2.22)	0.113
HDL-C (mmol/L)	1.25 ± 0.36	1.18 ± 0.35	1.11 ± 0.29^a^	<0.001
LDL-C (mmol/L)	3.07 ± 0.87	2.70 ± 0.93	2.80 ± 0.93	<0.001
hsCRP (mg/L)	1.52 (0.79, 2.71)	1.63 (0.89, 3.23)	1.93 (0.97, 3.90)	0.016
Medications, *n* (%)				
Statins	63 (31.7%)	261 (67.3%)	267 (73.4%)	<0.001
Aspirin	73 (36.7%)	277 (71.4%)	269 (73.9%)	<0.001
Calcium antagonists	29 (14.6%)	157 (40.5%)	117 (32.1%)^b^	<0.001
ARB/ACEI	28 (14.1%)	160 (41.2%)	156 (42.9%)	<0.001
Beta-blockers	54 (27.1%)	206 (53.1%)	181 (49.7%)	<0.001
Diuretics	18 (9.0%)	76 (19.6%)	63 (17.3%)	0.004

Abbreviations: CR—control; CP—calcified plaque; NCP—noncalcified plaque; MP—mixed plaque; BMI—body mass index; CAD—coronary artery disease; TC—total cholesterol; TG—triglycerides; HDL-C—high-density lipoprotein cholesterol; LDL-C——low-density lipoprotein cholesterol; hsCRP—high-sensitivity C-reactive protein; ARB—angiotensin II receptor blockers; ACEI—angiotensin-converting enzyme inhibitors. ^a^*P* < 0.01 for NCP/MP vs. CP. ^b^*P* < 0.05 for NCP/MP vs. CP.

**Table 2:** Demographic and clinical characteristics of the study subjects according to age-specific type of plaques.

Variables	Age < 60				Age ≥ 60			
	CR (*n* = 161)	CP (*n* = 159)	NCP/MP (*n* = 207)	*P*	CR (*n* = 38)	CP (*n* = 229)	NCP/MP (*n* = 157)	*P*
Age (years)	49.1 ± 7.1	53.2 ± 5.3	50.0 ± 5.8^a^	<0.001	64.0 ± 3.8	68.9 ± 6.0	68.0 ± 6.1	<0.001
BMI (kg/m^2^)	25.1 ± 3.5	26.2 ± 3.9	26.1 ± 3.8	0.009	23.8 ± 2.9	25.2 ± 3.4	25.0 ± 3.7^b^	0.070
Male, *n* (%)	78 (48.4%)	110 (69.2%)	154 (74.4%)	<0.001	11 (28.9%)	94 (41.0%)	89 (56.7%)	0.001
Hypertension, *n* (%)	74 (46.0%)	113 (71.1%)	140 (67.6%)	<0.001	18 (47.4%)	185 (80.8%)	116 (73.9%)	<0.001
Diabetes, *n* (%)	15 (9.3%)	34 (21.4%)	53 (25.6%)	<0.001	6 (15.8%)	62 (27.1%)	54 (34.4%)	0.053
Dyslipidemia, *n* (%)	61 (37.9%)	120 (75.5%)	166 (80.2%)	<0.001	16 (42.1%)	180 (78.6%)	133 (84.7%)	<0.001
Smoking, *n* (%)	37 (23.0%)	62 (39.0%)	88 (42.5%)	<0.001	2 (5.3%)	33 (14.4%)	38 (24.2%)^c^	0.005
Alcohol consumption, *n* (%)	41 (25.5%)	67 (42.1%)	93 (44.9%)	<0.001	0 (0.0%)	32 (14.0%)	26 (16.6%)	0.028
History of CAD, *n* (%)	24 (14.9%)	23 (14.5%)	28 (13.5%)	0.927	3 (7.9%)	16 (7.0%)	13 (8.3%)	0.891
Biochemical parameters								
TC (mmol/L)	4.94 ± 0.95	4.59 ± 1.09	4.62 ± 1.14	0.005	4.63 ± 1.36	4.45 ± 1.52	4.52 ± 1.31	0.729
TG (mmol/L)	1.46 (1.05, 2.14)	1.66 (1.13, 2.29)	1.62 (1.21, 2.41)	0.118	1.43 (0.95, 1.87)	1.39 (1.03, 1.83)	1.41 (1.05, 2.13)	0.380
HDL-C (mmol/L)	1.23 ± 0.37	1.13 ± 0.35	1.09 ± 0.28	<0.001	1.33 ± 0.34	1.22 ± 0.35	1.13 ± 0.30	0.001
LDL-C (mmol/L)	3.12 ± 0.85	2.82 ± 0.92	2.88 ± 0.93	0.008	2.87 ± 0.97	2.2.61 ± 0.93	2.70 ± 0.93	0.250
hsCRP (mg/L)	1.51 (0.73, 2.72)	1.68 (0.77, 3.40)	1.82 (0.81, 3.34)	0.279	1.84 (1.10, 2.71)	1.62 (0.93, 3.11)	2.26 (1.26, 4.63)^b^	0.003
Medications, *n* (%)								
Statins	52 (32.3%)	100 (62.9%)	150 (72.5%)	<0.001	11 (28.9%)	161 (70.3%)	117 (74.5%)	<0.001
Aspirin	60 (37.3%)	105 (66.0%)	145 (70.0%)	<0.001	13 (34.2%)	172 (75.1%)	124 (79.0%)	<0.001
Calcium antagonists	20 (12.4%)	55 (34.6%)	61 (29.5%)	<0.001	9 (23.7%)	102 (44.5%)	56 (35.7%)	0.025
ARB/ACEI	26 (16.1%)	58 (36.5%)	86 (41.5%)	<0.001	2 (5.3%)	102 (44.5%)	70 (44.6%)	<0.001
Beta-blockers	44 (27.3%)	75 (47.2%)	93 (44.9%)	<0.001	10 (26.3%)	131 (57.2%)	88 (56.1%)	0.002

Abbreviations: CR—control; CP—calcified plaque; NCP—noncalcified plaque; MP—mixed plaque; BMI—body mass index; CAD—coronary artery disease; TC—total cholesterol; TG—triglycerides; HDL-C—high-density lipoprotein cholesterol; LDL-C—low-density lipoprotein cholesterol; hsCRP—high-sensitivity C-reactive protein; ARB—angiotensin II receptor blockers; ACEI—angiotensin-converting enzyme inhibitors. ^a^*P* < 0.01 for NCP/MP vs. CP in subjects with age < 60 years. ^b^*P* < 0.01 for NCP/MP vs. CP in subjects with age ≥ 60 years. ^c^*P* < 0.05 for NCP/MP vs. CP in subjects with age ≥ 60 years.

**Table 3:** Demographic and clinical characteristics of the study subjects according to age-specific tertiles of hsCRP.

Variables	Age < 60				Age ≥ 60			
	<1.02 (*n* = 176)	1.02-2.37 (*n* = 176)	>2.37 (*n* = 175)	*P*	<1.25 (*n* = 141)	1.25–2.70 (*n* = 142)	>2.70 (*n* = 141)	*P*
Age (years)	50.5 ± 5.9	50.9 ± 6.3	50.7 ± 6.7	0.891	68.0 ± 5.9	67.4 ± 5.5	69.0 ± 6.5	0.058
BMI (kg/m^2^)	24.8 ± 3.5	26.3 ± 3.3	26.5 ± 4.2	<0.001	24.8 ± 3.3	25.1 ± 3.3	25.2 ± 3.9	0.617
Male, *n* (%)	111 (63.1%)	111 (63.1%)	120 (68.6%)	0.460	76 (53.9%)	56 (39.4%)	62 (44.0%)	0.044
Hypertension, *n* (%)	92 (52.3%)	117 (66.5%)	118 (67.4%)	0.005	102 (72.3%)	102 (71.8%)	115 (81.6%)	0.103
Diabetes, *n* (%)	25 (14.2%)	35 (19.9%)	42 (24.0%)	0.066	39 (27.7%)	46 (32.4%)	37 (26.2%)	0.488
Dyslipidemia, *n* (%)	108 (61.4%)	122 (69.3%)	117 (66.9%)	0.273	113 (80.1%)	107 (75.4%)	109 (77.3%)	0.624
TC (mmol/L)	4.64 ± 1.08	4.87 ± 1.03	4.62 ± 1.10	0.056	4.37 ± 1.74	4.74 ± 1.28	4.36 ± 1.19	0.041
TG (mmol/L)	1.41 (0.96, 2.11)	1.67 (1.31, 2.41)	1.64 (1.09, 2.49)	0.118	1.28 (0.95, 1.64)	1.51 (1.12, 2.15)	1.41 (1.00, 2.06)	0.380
LDL-C (mmol/L)	2.85 ± 0.87	3.05 ± 0.89	2.90 ± 0.95	0.091	2.53 ± 0.93	2.85 ± 0.91	2.62 ± 0.94	0.011
HDL-C (mmol/L)	1.24 ± 0.35	1.16 ± 0.33	1.04 ± 0.28	<0.001	1.24 ± 0.32	1.24 ± 0.32	1.11 ± 0.34	0.001
CCTA data, *n* (%)								
CR	54 (30.7%)	61 (11.6%)	46 (26.3%)	0.234	11 (7.8%)	18 (12.7%)	9 (6.4%)	0.151
CP	53 (30.1%)	44 (25.0%)	62 (35.4%)	0.104	92 (65.2%)	68 (47.9%)	69 (48.9%)	0.005
NCP/MP	69 (39.2%)	71 (40.3%)	67 (38.3%)	0.925	38 (27.0%)	56 (39.4%)	63 (44.7%)	0.007

Abbreviations: hsCRP—high-sensitivity C-reactive protein; BMI—body mass index; TC—total cholesterol; TG—triglycerides; HDL-C—high-density lipoprotein cholesterol; LDL-C—low-density lipoprotein cholesterol; CCTA—coronary computed tomography angiography; CR—control; CP—calcified plaque; NCP—noncalcified plaque; MP—mixed plaque.

**Table 4:** Correlation between hsCRP and other cardiovascular risk factors.

Variables	Age < 60 years (*n* = 527)		Age ≥ 60 years (*n* = 424)	
	*r*	*P*	*r*	*P*
Age (years)	0.055	0.209	0.065	0.181
BMI (kg/m^2^)	0.173	<0.001	0.055	0.256
TC (mmol/L)	-0.011	0.809	0.015	0.752
TG (mmol/L)	0.132	0.002	0.082	0.091
HDL-C (mmol/L)	-0.258	<0.001	-0.203	<0.001
LDL-C (mmol/L)	0.003	0.941	0.009	0.854

Abbreviations: hsCRP—high-sensitivity C-reactive protein; BMI—body mass index; TC—total cholesterol; TG—triglycerides; HDL-C—high-density lipoprotein cholesterol; LDL-C—low-density lipoprotein cholesterol.

**Table 5:** Regression analysis to assess the presence of NCP/MP according to hsCRP^a^.

Variables	Age < 60 years		Age ≥ 60 years	
	OR (95% CI)	*P*	OR (95% CI)	*P*
Presence of atherosclerosis plaques				
hsCRP	1.074 (0.999–1.155)	0.053	1.032 (0.921–1.032)	0.588
hsCRP tertiles				
Tertile 1	1		1	
Tertile 2	0.574 (0.339–0.972)	0.039	0.483 (0.186–1.251)	0.134
Tertile 3	0.970 (0.556–1.693)	0.915	1.133 (0.287–3.321)	0.819
The presence of CP				
hsCRP	1.032 (0.975–1.093)	0.279	0.932 (0.882–0.986)	0.014
hsCRP tertiles				
Tertile 1	1		1	
Tertile 2	0.650 (0.393–1.077)	0.094	0.445 (0.269–0.736)	0.002
Tertile 3	1.059 (0.645–1.738)	0.821	0.416 (0.250–0.692)	0.001
Presence of NCP/MP				
hsCRP	1.018 (0.961–1.078)	0.538	1.091 (1.031–1.154)	0.002
hsCRP tertiles				
Tertile 1	1		1	
Tertile 2	0.944 (0.593–1.503)	0.809	2.110 (1.235–3.605)	0.006
Tertile 3	0.889 (0.553–1.430)	0.629	2.805 (1.639–4.802)	<0.001

Abbreviations: hsCRP—high-sensitivity C-reactive protein; CR—control; CP—calcified plaque; NCP—noncalcified plaque; MP—mixed plaque. ^a^Adjusted for age, sex, BMI, hypertension, diabetes, dyslipidemia, smoking, statin, aspirin, and ARB/ACEI.

## Data Availability

The clinical and laboratory data used to support the findings of this study are available from the corresponding author upon request.
